# Injection of Uric Acid

**Published:** 1873-06

**Authors:** 


### Injection of Uric Acid.

M. Gigot-Suard submitted to the Academy of Medicine, on the 21st of January, an interesting paper on this subject, of which we give a summary. Administered to dogs in doses of from twenty centigrammes to four grammes, in twenty-four hours, for a period varying from one to two months, uric acid produces morbid symptoms which are very remarkable and which may help to clear up the pathogeny of many chronic diseases. In several instances the alkalinity of the serum of the blood was diminished to the point of neutrality. The microscope and chemical analysis revealed the presence of crystals of uric acid, of oxalic acid, and of urate of soda. The organs and tissues on which the acid has shown its action are, in order of frequency: the skin, the mucous membranes and their glands, the lungs, the kidneys, the liver, the pancreas, the brain, the lymphatic glands, the articulations, the spleen, the pericardium, the spinal and cerebral meninges, and the heart.

The *skin* exhibited almost all the alterations described by dermatologists — erythema, papulæ, vesiculæ, pustulæ, and squamæ. The *mucous membranes* were more or less strongly congested, rarely softened. Most often affected were the membranes of the mouth, nose, eyes, and bronchi. The glands were hypertrophied and even ulcerated; but the last lesion was commonest in the glands and tubules of the rectum. The *lungs* were congested and hepatized, with or without apoplectic centres, to say nothing of tuberculization, which will be noticed presently. The maladies of the kidneys varied from simple congestion of the cortical substance to the characteristics of Bright's disease. The *liver* was several times congested, and once showed fatty degeneration. In the *pancreas* nothing was observed except a more or less extensive injection in its surface. The same was true of the *brain* where the injection affected only the surface and part of the gray substance. Cancerous and tuberculous degeneration appeared several times in the *lymphatic glands*. In other cases there was merely engorgement. In the *articulations* the only lesions remarked were, increase of the synovial fluid, deepening of the color of the cartilages, and injection of the synovial membranes. There were no deposits of urate of soda. Lesions of the spleen were rare, and consisted of nothing but injection and slight increase of color in some spots. In one case the spinal meninges and the pericardium were strongly injected. The *heart* was affected in but one case, the walls being enormously thickened, and the endocardium having a silvery look. Besides these organic lesions, there were, in one case, symptoms of diabetes; in three cases, pulmonary tuberculosis; in one, a woody scirrhous tumor of the skin on the neck; and in one an epithelioma on the tongue.—(*Gazette Hebdomadaire, January.*) — *N. Y. Medical Journal.*

